# Study on the properties of modified octadecyltrichlorosilane (OTS) anti-relaxation coatings for cesium atomic cell: a molecular dynamics simulation

**DOI:** 10.1039/d5ra01627d

**Published:** 2025-08-28

**Authors:** Hao Zhai, Hengjiao Gao, Wei Li

**Affiliations:** a School of Instrumentation and Optoelectronic Engineering, Beihang University Beijing 100191 China liwei02@buaa.edu.cn; b Science and Technology on Vacuum Technology and Physics Laboratory, Lanzhou Institute of Physics Lanzhou 730000 Gansu Province China gaohengjiao@163.com; c Gansu Natural Energy Institute Lanzhou 730046 Gansu Province China; d Hefei National Laboratory Hefei 230088 Anhui Province China; e Institute of Large-Scale Scientific Facility and Centre for Zero Magnetic Field Science, Beihang University Beijing 100191 China; f National Institute of Extremely Weak Magnetic Field Infrastructure Hangzhou 310051 Zhejiang Province China

## Abstract

To investigate the anti-relaxation performance of FOTS-modified OTS coatings on the inner walls of cesium (Cs) atomic cell, this study employs molecular dynamics (MD) simulations to explore the self-assembly process of FOTS-modified OTS molecular chains on the SiO_2_ (001) surface and evaluates the effects of FOTS chain amounts, water molecule content, and temperature on the diffusion behavior of Cs atoms. Results show that the optimized interface model of the FOTS-modified OTS coating and SiO_2_ substrate achieves thermodynamic and energetic equilibrium under the conditions of 25 °C and 2000 ps. The film formation process of FOTS-modified OTS chains on SiO_2_ surfaces involves three distinct stages: initial anchoring, conformational rearrangement, and structural relaxation and equilibrium configuration. The molecular chains evolve from an initial perpendicular orientation into a final stable configuration parallel to the substrate surface. Increasing the FOTS content effectively reduces the diffusion coefficient of Cs atoms. The optimal OTS : FOTS blending ratio is identified as 20 : 8, yielding a minimum diffusion coefficient of 0.204 × 10^−6^ cm^2^ s^−1^. Water molecules exhibit a significant influence on Cs diffusion dynamics. As the water content increases from 1 to 20, the diffusion coefficient rises from 0.271 × 10^−6^ cm^2^ s^−1^ to 2.387 × 10^−6^ cm^2^ s^−1^. Additionally, the mobility of Cs atoms displays a non-monotonic dependence on temperature, where the diffusion coefficient initially decreases and then increases with rising temperature, reaching a minimum value of 0.172 × 10^−6^ cm^2^ s^−1^ at 80 °C. These findings provide theoretical guidance for the design, optimization, and fabrication of high-performance anti-relaxation coatings for Cs atomic vapor cell applications.

## Abstract 2

The effects of FOTS chains amounts, water molecule content, and temperature on the diffusion behavior of Cs atoms were studied by molecular dynamics simulations method.

## Graphical abstract

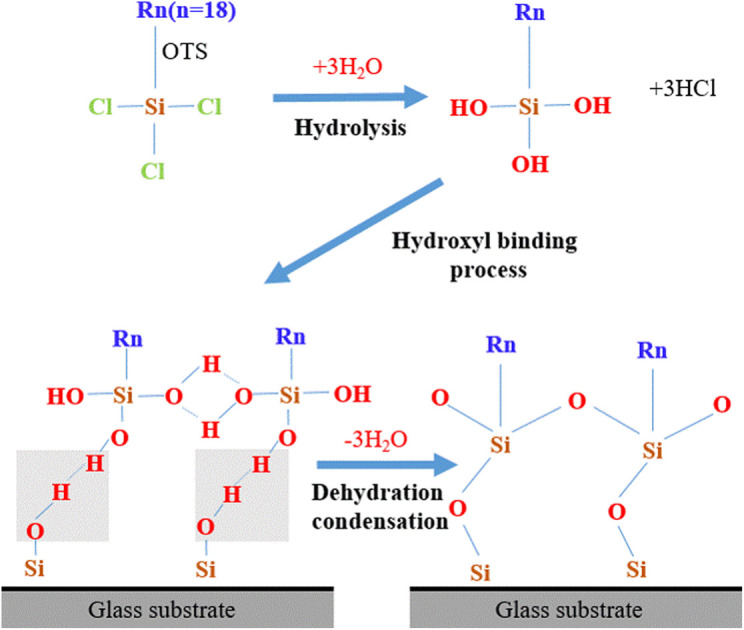

## Introduction

A magnetometer serves as the core sensor in unmanned aerial vehicle (UAV) aeromagnetic survey systems, playing a pivotal role in determining the measurement accuracy.^1,2^ Current magnetometer technologies include fluxgate, optically pumped, proton precession, superconducting, and atomic magnetometers. Among these, the coherent population trapping (CPT) atomic magnetometer measures magnetic fields by exploiting the Zeeman splitting of atomic energy levels.^3^ Specifically, the CPT atomic magnetometers operate based on the coherent population trapping effect and the Zeeman splitting phenomenon in atomic fine-structure energy levels under magnetic fields.^4^ This technology exhibits superior long-term stability, high accuracy, omnidirectional sensitivity (eliminating orientation blind zones), and compact coil-free probe designs.^5–7^ Compared with other magnetometers, CPT atomic magnetometers are uniquely suited for small UAV aeromagnetic applications due to their miniaturization potential and robust performance in dynamic environments. As a pivotal component in quantum technologies, cesium atomic cells play an indispensable role in precision measurement and fundamental scientific research. However, persistent challenges, such as atom-wall relaxation (quantum decoherence induced by atom–surface interactions), environmental noise (thermal and magnetic fluctuations), and miniaturization bottlenecks (trade-offs between vacuum integrity and compact design), remain critical research frontiers.^8,9^ Future advancements necessitate innovations in materials science, breakthroughs in fabrication techniques, and interdisciplinary convergence to propel these systems toward enhanced performance, reduced costs, and expanded applicability in next-generation quantum sensors, spaceborne atomic clocks, and miniaturized quantum inertial navigation systems. Despite its numerous advantages, the sensitivity of CPT atomic magnetometers still requires further enhancement to achieve applications under more demanding environmental conditions, including mechanical vibration and shock, high and low temperature variations, spatial electromagnetic interference, vacuum environments, chemical corrosion/contamination, and space radiation.

The performance of atomic cells plays a critical role in determining the sensitivity of CPT magnetometers. Currently, the evaluation of atomic cell performance is achieved by measuring the CPT signal spectral line, with the key evaluation metrics being the CPT signal amplitude and linewidth. To prolong the service life of cesium atomic cells, anti-relaxation coatings are typically applied to the inner walls or buffer gases are introduced into the alkali metal atomic cells.^10^ Among these approaches, the implementation of anti-relaxation coatings constitutes a critical technical pathway for enhancing the sensitivity of CPT atomic magnetometers. Its core mechanism lies in suppressing inelastic collisions between atoms and the cell walls, thereby extending atomic coherence time, narrowing CPT resonance linewidth, and ultimately improving magnetic detection sensitivity. Compared to the buffer gas method, anti-relaxation-coated atomic cells exhibit the following key advantages: elimination of additional gas-induced spectral broadening, enabling ultimate linewidth compression, enhanced signal intensity through reduced decoherence and superior miniaturization and integration compatibility.^11^ However, the fabrication processes for alkali metal atomic cells with such coatings remain immature, resulting in significant batch-to-batch variability among cells produced under identical conditions.^12,13^ Therefore, advancing research on anti-relaxation coatings for cesium atomic cells is of paramount significance. The earliest materials employed for anti-relaxation coatings were straight-chain alkanes, with paraffin (a mixture of long-chain n-alkanes, typically C_20_–C_40_) being the most representative.^14^ In alkali metal atomic cells coated with paraffin, alkali atoms can undergo up to 10 000 collisions with the inner walls without depolarization. However, paraffin coatings exhibit a critical limitation: they melt at 60–80 °C, restricting their utility to low-temperature regimes (≤80 °C).^15^ The melting points of n-alkanes increase with molecular weight, for instance, hexacontane (C_60_H_122_) and ultra-long-chain hydrocarbons (*e.g.*, C_390_H_782_, the longest reported hydrocarbon) have melting points exceeding 100 °C.^16^ There are no studies that have documented their application in anti-relaxation coatings to date. To address the demand for high-temperature-resistant coatings (operable >80 °C), organochlorosilanes have emerged as promising alternatives. Among these, the octadecyltrichlorosilane (OTS) has been extensively investigated. Recent studies demonstrate that potassium-filled OTS-coated cells achieve operational stability at temperatures up to 120 °C, with potassium atoms sustaining up to 2100 collisions before depolarization—a significant advancement for high-temperature quantum sensing applications, such as aerospace inertial navigation and deep-well magnetometry.^17–19^ To address the inherent limitations of conventional OTS anti-relaxation coatings under high-temperature, corrosive environments, and dynamic mechanical loads, structural modification of OTS coatings is imperative. Fluorination modification, which introduces fluorine atoms or fluorinated functional groups (*e.g.*, –CF_3_, –CF_2_–), endows OTS coatings with superhydrophobicity (contact angle >160°), enhanced thermal stability (operable up to 170 °C), superior corrosion resistance (tolerant to 98% H_2_SO_4_), and improved mechanical robustness.^20,21^ Current research on anti-relaxation coatings for cesium atomic cells faces two major challenges. First, the development of coating systems with outstanding anti-relaxation performance and efficient fabrication techniques. Second, the ability of such coating systems to maintain their anti-relaxation properties at elevated temperatures (>80 °C). To overcome these challenges, this study introduces a perfluorooctyltrichlorosilane (FOTS)-modified OTS coating system that capitalizes on the advantageous properties of fluorinated chains, including low surface energy, excellent thermal stability, and strong hydrophobicity. This innovative design effectively suppresses cesium atom diffusion at high temperatures, thereby significantly enhancing the anti-relaxation performance of the coating to meet more demanding application requirements. However, the underlying mechanisms governing fluorinated OTS coatings—such as fluorine-induced electronic shielding, Si–O–Si–F network dynamics, and interfacial adhesion—remain poorly elucidated. Experimental investigations into these mechanisms are time-intensive and resource-demanding, while the practical efficacy of fluorinated coatings in cesium cells remains challenging to predict empirically. By integrating computational insights with targeted experiments, researchers can bypass costly trial-and-error approaches, accelerating the development of next-generation anti-relaxation coatings.^22^ For instance, molecular dynamics simulations have revealed that fluorinated OTS coatings achieve a 40% reduction in Cs atom adsorption probability compared to non-fluorinated counterparts, aligning closely with experimental QCM measurements (±5% deviation). This paradigm shift toward computational-experimental synergy not only enhances fundamental understanding but also paves the way for scalable, high-performance coatings tailored to extreme operational conditions.

Therefore, we developed an innovative FOTS-modified OTS anti-relaxation coating system that substantially enhances both hydrophobicity and high-temperature resistance of the coating and applied the molecular dynamics simulation method to investigate the performance of fluorinated OTS anti-relaxation coatings in this work. The self-assembly process of FOTS-modified OTS polymer chains can be elucidated from a molecular perspective, providing fundamental insights into the coating formation mechanism. By systematically examining the OTS-substrate interfacial properties, and the effects of fluorinated chain modification amounts, water molecule content and temperature on the diffusion behavior of cesium atoms within the OTS coatings, we aim to clarify the influence of synthesis parameters on OTS coating performance. These insights will establish a predictive framework to guide the fabrication and optimization of fluorinated OTS coatings for practical applications, particularly in cesium cells requiring enhanced thermal stability and minimized atom-wall relaxation.

## Film formation mechanism, modeling and calculation methods

### Film formation mechanism

**Fig. 1:**
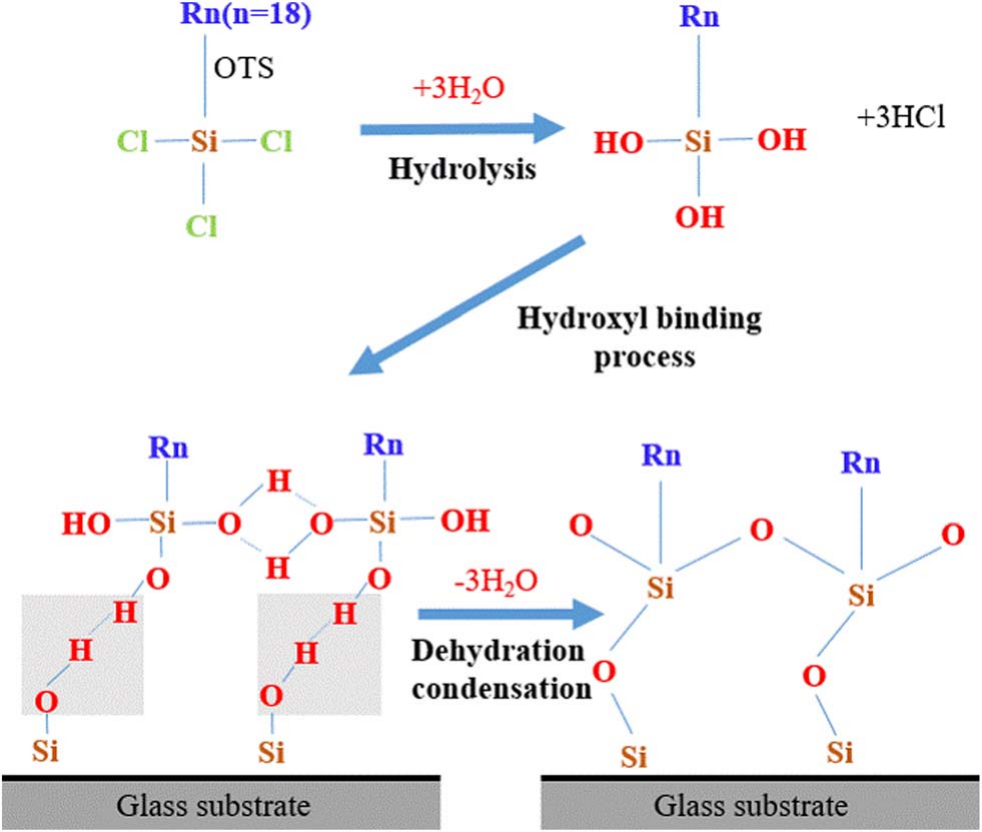
Schematic of the film forming process of OTS molecule on glass substrate.

The film formation process of OTS molecules on the glass substrate is as follows: First, the OTS molecules undergo hydrolysis, that is, the three Cl atoms at the end of the OTS molecule react with water to form hydrogen chloride (HCl) molecules, while the three Cl atoms at the end of the OTS molecule are replaced by hydroxyl groups to form silanol groups (Si–OH), and the silanol groups undergo dehydration and condensation reactions with the hydroxyl groups on the glass substrate.^23^ It is bonded to the glass substrate in the form of covalent silico–oxygen bonds. In addition, dehydration and condensation reactions occur between hydrolyzed silanol groups to form an ordered and oriented single-layer coating. The film formation process of OTS molecules on the glass substrate is shown in Fig. 1.

At present, the most common approach for preparing OTS anti-relaxation coatings is the solution synthesis method. The actual experimental environment and conditions significantly influence the film formation process of OTS molecules. All of the factors, such as substrate surface state, number of modified perfluoroalkyl chains, temperature, water content in the system, immersion time, OTS solution concentration, solvent type, and aging time, have a substantial impact on the growth process and performance of OTS coatings. In subsequent studies, we plan to introduce the vacuum deposition technology to prepare these anti-relaxation coatings, especially atomic layer deposition(ALD) technology, which can effectively eliminate the influence of multiple process variables, including solvent selection, immersion and aging time and solution concentration. Therefore, this study mainly focuses on the effects of fluorinated chain quantity, water molecule content, and synthesis temperature on coating performance.

### Modeling

The modeling procedures were implemented using the Visualizer and Amorphous Cell modules in Materials Studio 2019. The establishment of the SiO_2_ substrate/perfluorinated-chain-modified OTS coating interface model involved three key steps: (1) Molecular model construction: Development of individual component models, including modified perfluorinated chains, octyltrichlorosilane (OTS) chains, and hydroxylated SiO_2_ substrate surfaces. (2) Coating assembly: Generation of the perfluorinated-chain-modified OTS coating structure through molecular packing optimization under periodic boundary conditions that replicate the simulated material system infinitely in three-dimensional space, forming a virtually extended system to eliminate boundary effects and make the simulation results closer to the real material properties. Under periodic boundary conditions, the types and total number of particles in the unit cell remain unchanged. When a particle crosses one boundary of the unit cell, it re-enters from the opposite boundary. To avoid non-physical interactions between atoms and their own mirror images, the cutoff radius must be set to less than half of the smallest box edge length. In this study, it was set to 14 Å. (3) Interface cross-linking simulation: Molecular dynamics (MD) simulation of the curing process to model Si–O–Si covalent bond formation between OTS molecules and the SiO_2_ substrate, with energy minimization and NVT ensemble equilibration. The specific steps are as follows.

**Fig. 2:**
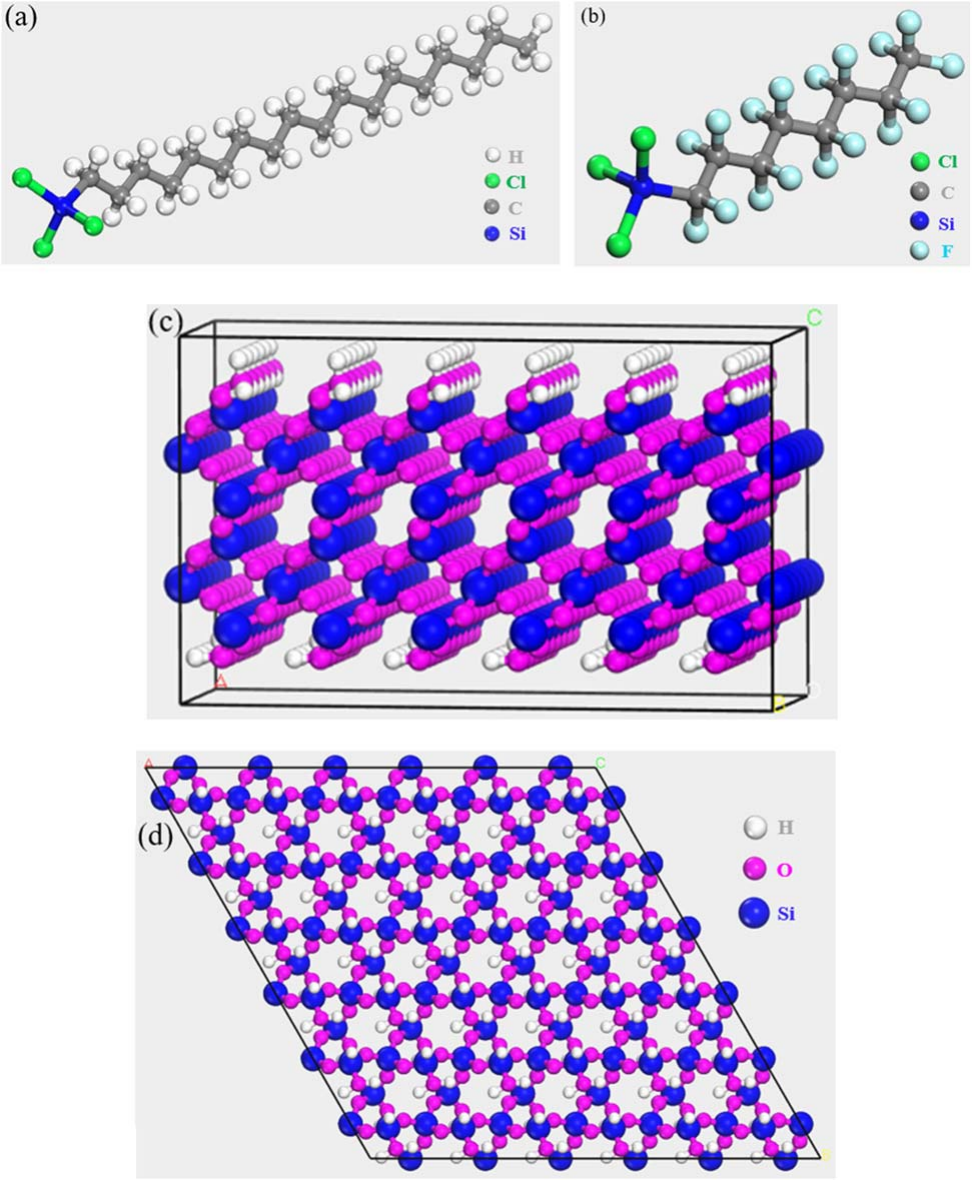
Structure of OTS molecule, FOTS molecule and substrate surface: (a) OTS molecule; (b) FOTS molecule; (c) substrate surface from the side view; (d) substrate surface from the top view.

The molecular structures of OTS and FOTS chains were independently sketched using the Visualizer module, with optimized geometries shown in Fig. 2(a) and (b), respectively. The unit cell of a silica crystal was initially exported from the structural database, followed by cleaving the (001) surface. The lattice parameters of silica are *a* = *b* = 4.91 Å, *c* = 5.41 Å, with *α* = *β* = 90° and *γ* = 120°. As the hydroxyl groups were concurrently transported to the surface during the thermal treatment process, oxygen atoms on the silica surface were passivated with hydrogen atoms to form hydroxyl groups. The SiO_2_(001) surface model was expanded into a 6 × 6 × 3 supercell, as shown in Fig. 2(c) and (d) from the side and top view, with final dimensions of 29.48 Å × 29.48 Å × 15.32 Å. The supercell model contains 864 atoms in total, including 144 hydrogen atoms, 504 oxygen atoms, and 255 silicon atoms. To obtain amorphous silica, simulated annealing was performed on the current structure. Specifically, five cyclic annealing processes were conducted under the NPT ensemble, with the temperature oscillating between 300 K and 500 K. Subsequently, a three-dimensional periodic lattice containing 20 octyltrichlorosilane (OTS) molecules and 8 perfluorooctyltrichlorosilane (FOTS) molecules was constructed and the system density was initialized at 1.2 g cm^−3^. We constructed modified OTS coating models containing cesium atoms and water molecules by sequentially adding varying quantities of cesium atoms (30), FOTS molecular chains (0, 4, 8, 12, 16, 20), and water molecules (1, 5, 10, 15, 20) using the Amorphous Cell Calculation module of the software.

**Fig. 3:**
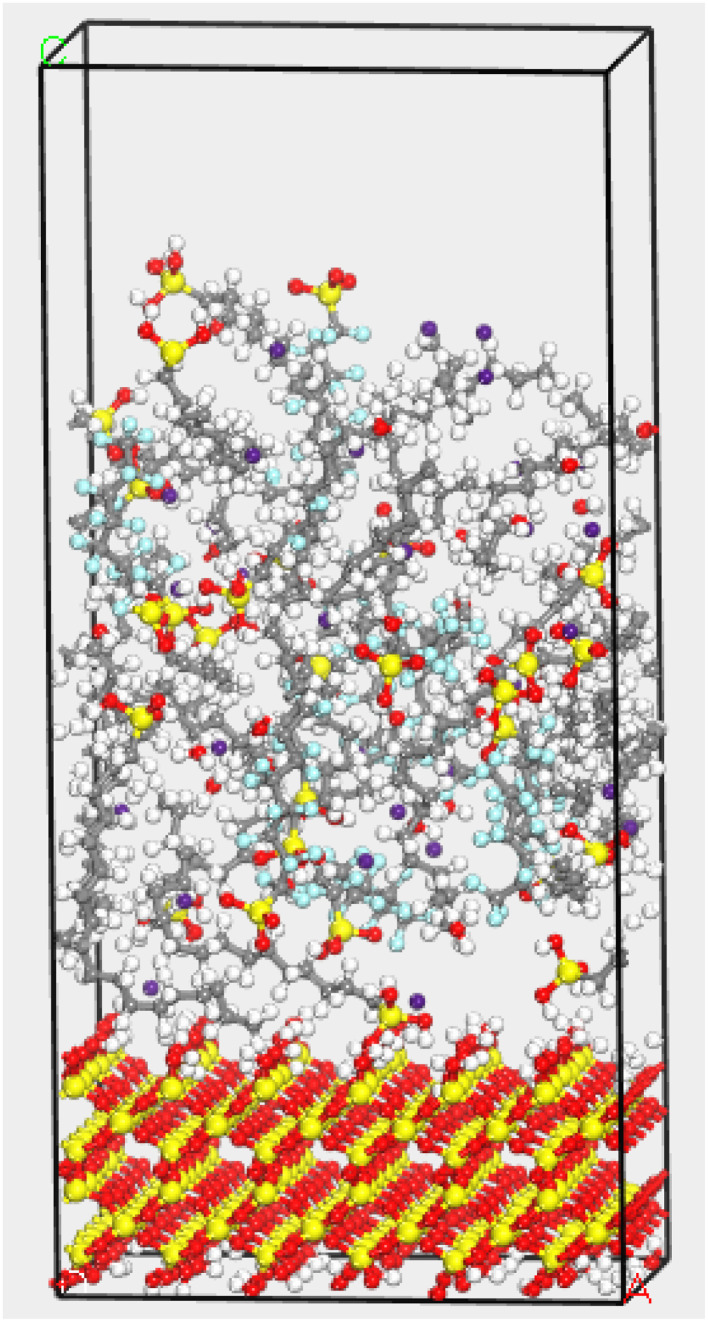
Interface model of fluorinated modified OTS coating/SiO_2_(001) including the Cs atoms and H_2_O molecules.

Finally, the established SiO_2_(001) substrate surface model and modified OTS coating model were assembled into a bilayer structure. Here, Layer 1 was defined as the SiO_2_(001) substrate surface, and Layer 2 was designated as the uncrosslinked modified OTS coating, with the vacuum layer thickness set to 15 Å. Structural optimization was first performed on the bilayer model to obtain the most stable, lowest-energy configuration. The final modified OTS coating/SiO_2_(001) interface model, including the Cs atoms and H_2_O molecules, is illustrated in Fig. 3. Subsequently, simulations of the modified OTS coating cross-linking process were conducted. Cross-linking refers to the junction points formed between molecular chains through covalent bonds, hydrogen bonds, ionic interactions, or other intermolecular forces. In practical polymerization processes of polymeric materials, the cross-linking density generally fails to reach the theoretical maximum (100%) due to multifactorial constraints, including thermodynamic equilibrium limitations, kinetic barriers, steric hindrance effects, and practical processing restrictions. The predetermined cross-linking degree denotes the predetermined proportion of cross-links artificially incorporated into the simulation system that effectively replicates the structural characteristics of chemically cross-linked networks in real material systems. The cross-linking reaction algorithmically terminates upon reaching the predetermined cross-linking degree (set as 90% in this study). During the simulation, the distance between the oxygen atoms at the head groups of OTS/FOTS and the hydroxyl oxygen atoms on the substrate surface was constrained to a range of 0.3–1.0 nm. Reactions were considered feasible if the atomic distances were within this range, and the process continued until the predetermined cross-linking degree was achieved. Cross-linking condensation reactions were simulated using an in-house cross-linking reaction program to form bonds. For each cutoff distance condition, the resulting cross-linked modified OTS coating models underwent structural optimization to ensure reasonable bond lengths and stable molecular configurations.

### Calculation methods

Before the molecular dynamics simulation, the structure of the interface was optimized to minimize its energy and stabilize the structure. The dynamics simulation process was performed using the Forcite Plus module. The simulation includes the following three parts. Firstly, in order to fully relax the atoms in the material model and reduce the internal stress of the coating, it is necessary to conduct an annealing simulation for each layered and interface model. The initial heating temperature and maximum temperature were set as 300 K and 500 K, respectively, with a temperature change rate of 5 K per cycle. The annealing process was performed for 5 cycles, with each cycle consisting of 500 molecular dynamics steps. The convergence criteria required the standard deviations of energy, temperature, and pressure to be less than 5%. Secondly, the NVT ensemble is employed during the pre-equilibration stage for systems requiring fixed density, such as liquid-phase systems. Since the model is constructed layer-by-layer and subsequently superimposed to form an interface model, individual layers may initially exhibit unrealistic atomic positions or bond lengths. The NVT ensemble is thus used to optimize the initial configuration by rapidly relaxing the energy of the system to achieve an equilibrated state under ambient temperature and pressure conditions. Therefore, the NVT (canonical ensemble, constant Number–Volume–Temperature) ensemble was performed at a temperature of 298 K to enable the molecules and atoms in the system to obtain enough kinetic energy that can eliminate agglomeration between atoms in the simulation process. The removal of atom agglomeration was primarily based on the following considerations. (1) The polymerization process represents an equilibrium state where functional groups of polymer chains form bonds under optimized conditions. Artificial agglomeration may be misinterpreted as phase separation, nucleation, or interfacial phenomena, which would hinder normal atomic diffusion and vibration. Such artifacts can significantly alter relaxation processes, reaction pathways, and other kinetic properties. Therefore, removal of these unphysical aggregation phenomena is required to maintain consistency with actual experimental conditions. (2) Abnormal atomic agglomeration typically stems from either excessively strong short-range attractive interactions in the force field or improper parameterization, resulting in unrealistically small interatomic distances. This contradicts the equilibrium structural characteristics observed in real material systems (*e.g.*, liquids or solid solutions). (3) Regarding computational costs in simulations, when atoms aggregate, the strong interatomic forces necessitate extremely short time steps in Verlet and similar algorithms, significantly increasing computational expenses. Moreover, at excessively close distances, the repulsive force term grows precipitously, resulting in instability of the total system energy and potentially causing simulation failure. Thirdly, the (isothermal–isobaric ensemble, constant Number–Pressure–Temperature) ensemble is thus used to optimize the initial configuration by rapidly relaxing the energy of the system to achieve an equilibrated state under ambient temperature and pressure conditions. Subsequently, simulations using the NPT ensemble are performed to first adjust the system density to a stable value approximating experimental density, followed by pressure and temperature regulation to mimic real-world environmental conditions as closely as possible. Therefore, the NPT ensemble simulations at 298 K and 1 atm were carried out to calculate and analyze the related performance of the fluorinated modified OTS coating-substrate system.^24^ The COMPASS force field was used in the simulation process and the simulation accuracy was considered acceptable when the integration time step, cutoff radius, electrostatic interactions, van der Waals force and energy minimization convergence criteria were set as 1.0 fs, 1.4 nm, Ewald, atom-based and less than 100 kJ mol^−1^, respectively. The force field parameters for the cesium atom are as follows: van der Waals radius (*σ*_Cs_) = 4.65 Å, minimum potential well depth (*ϵ*_Cs_) = 0.04 kcal mol^−1^, and charge (*q*_Cs_) = 0 *e*. During energy relaxation and equilibration process of the modified OTS anti-relaxation coating and silica substrate surface models, the simulation time and step size were set as 200 ps and 1 ps, respectively. While for the fluorinated modified OTS coating-substrate system, the corresponding simulation time and step size were set to 2000 ps and 1 ps, respectively. The temperature and pressure of the interface system were adjusted using the Andersen thermostat and Berendsen controller in the simulation process.^25^

During the simulation, the dynamic behavior of cesium atoms in various coating systems was investigated through mean square displacement (MSD) analysis. This metric statistically quantifies the temporal evolution of particle displacements by measuring the squared distance between the position of the particle at time *t* [*R*_i_(*t*)] and its initial position [*R*_i_(0)], as defined by the following equation.^26^1
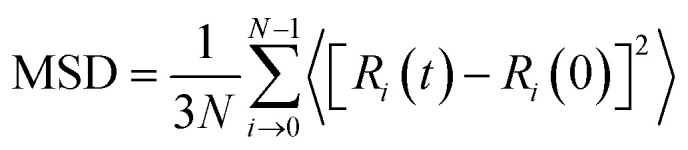
*N*, *R*_*i*_(*t*) and *R*_*i*_(0) denote the total number of selected particles in the system, the position vectors of the *i* particle at time *t* and the initial reference time (*t* = 0), respectively.

## Results and discussions

### System equilibrium and self-assembly process of OTS chains in coatings

**Fig. 4:**
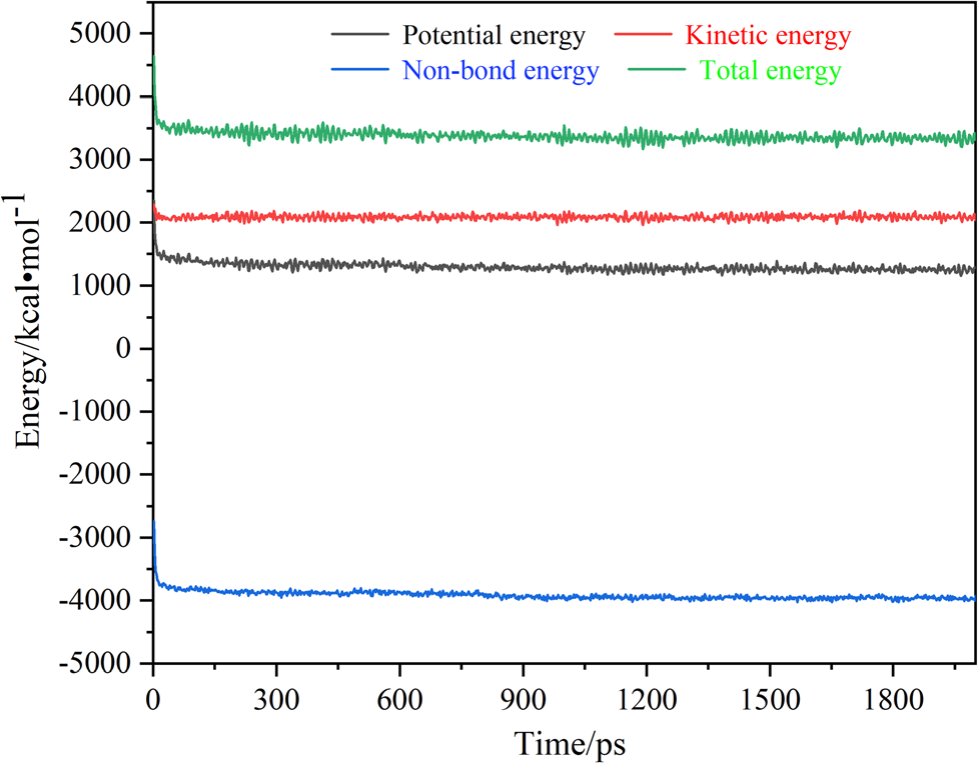
Energy evolution curves of the interface model of SiO_2_/OTS-FOTS during the dynamic equilibrium process.

The total energy of the system typically comprises kinetic energy, potential energy, and non-bonded interaction energy. The kinetic energy refers to the total energy arising from the continuous motion of all atoms within the modified OTS coating/SiO_2_(001) system. The potential energy represents the total energy associated with the positions of all atoms. The non-bonded interaction energy includes contributions from electrostatic interactions, van der Waals forces, and hydrogen bonding between atoms. The total energy of the system is the sum of these three energy components. The energy evolution curves during the dynamic equilibrium process are shown in Fig. 4. It can be seen that all three energy components (kinetic, potential, and non-bonded) exhibited significant fluctuations during the initial relaxation phase (within ∼50 ps). Subsequently, this system reached a dynamic equilibrium state, where its energy variations stabilized into minor oscillations within a narrow range before converging to steady plateau values. Under the NPT ensemble conditions (298 K, 2000 ps simulation duration), the system demonstrated stabilized energy fluctuations within an extremely narrow margin (<0.5% relative variation). This confirms that the interface system has reached a fully structural relaxation state, with atomic motions reaching steady-state behavior and internal stresses achieving dynamic balance. This fully equilibrated interface model thus provides a reliable foundation for subsequent investigations of cesium diffusion behavior in modified OTS coatings under controlled conditions. Therefore, all subsequent computational analyses of Cs atom diffusion pathways in modified OTS coatings were performed using these validated equilibrium parameters to ensure thermodynamic consistency.

**Fig. 5:**
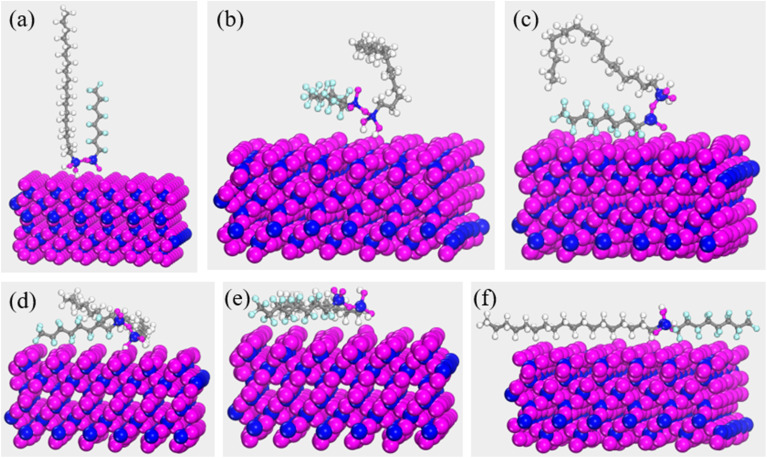
Self-assembly dynamics of FOTS-modified OTS chains on the SiO_2_ substrate surface: (a) 0 ps; (b) 50 ps; (c) 300 ps; (d) 1000 ps; (e) 1500 ps; (f) 2000 ps.

Fig. 5 delineates the self-assembly dynamics of FOTS-modified OTS chains on the SiO_2_(001) substrate surface, with initial Si atoms oriented vertically downward. The molecular evolution proceeds through three distinct phases. First is the initial anchoring stage. The terminally bonded oxygen atoms (coordinated to silicon) at the FOTS-OTS chain heads establish primary contacts with surface oxygen atoms of the SiO_2_ substrate (as shown in Fig. 5(a)–(c)). Concurrently, fluorinated segments within the FOTS chains approach the substrate interface, inducing a gradual chain reorientation toward parallel alignment. Second is the conformational rearrangement state (as shown in Fig. 5(d) and (e)). Driven by chain tension effects, the hydrocarbon backbones gradually migrate toward the substrate. This process induces molecular chain twisting accompanied by length contraction, while the hydroxyl groups between adjacent chains undergo condensation reactions, ultimately forming the Si–O–Si bridging bonds. Third is the structural relaxation and equilibrium configuration state. Following the completion of condensation reactions between hydroxyl groups, the molecular chains undergo structural relaxation through torsion and oscillation, ultimately attaining a stable configuration parallel to the substrate surface (as shown in Fig. 5(f)). This multistage assembly mechanism confirms that the silicon-terminated headgroups govern the interfacial binding hierarchy, providing critical insights for designing OTS-based anti-relaxation coatings.

### Effect of FOTS amounts on the relaxation properties of cesium atoms

The modification mechanism of OTS coatings by FOTS chains involves the preferential condensation reaction between the head silanol groups (–SiOH) of FOTS and OTS chains. This reaction forms a three-dimensional Si–O–Si cross-linked network. Subsequently, the newly generated active silanol groups undergo further condensation with hydroxyl groups (–OH) on the substrate surface, forming covalent bonds (Si–O–Si) to achieve chemical anchoring. It is crucial to calculate the diffusion coefficient of cesium atoms in the coating, as it serves as one of the key indicators for evaluating the performance of anti-relaxation coatings. This importance stems from the following primary reasons. First, a low diffusion rate helps maintain the lattice integrity of the anti-relaxation coating, preventing phase transitions or grain boundary migration and ensuring structural stability. Second, a low diffusion coefficient restricts the positional fluctuation amplitude of cesium atoms to below *λ*/20(*λ* = 852 nm), thereby helping to preserve their spin-polarized state as much as possible and extending the lifetime of the cesium atomic vapor cell. Third, it contributes to the chemical stability of the cesium atomic cell surface. A low diffusion coefficient reduces the likelihood of redox reactions, such as Cs + SiO_2_ → Cs_2_O + Si, thereby minimizing cesium consumption. Therefore, this section investigates the influence of the FOTS chain on the diffusion process of cesium atoms. The number of OTS chains is fixed at 20, while the number of FOTS chains is set to 0, 4, 8, 12, 16, and 20, corresponding to the OTS : FOTS ratios of 20 : 0, 20 : 4, 20 : 8, 20 : 12, 20 : 16, and 20 : 20, respectively.

**Fig. 6:**
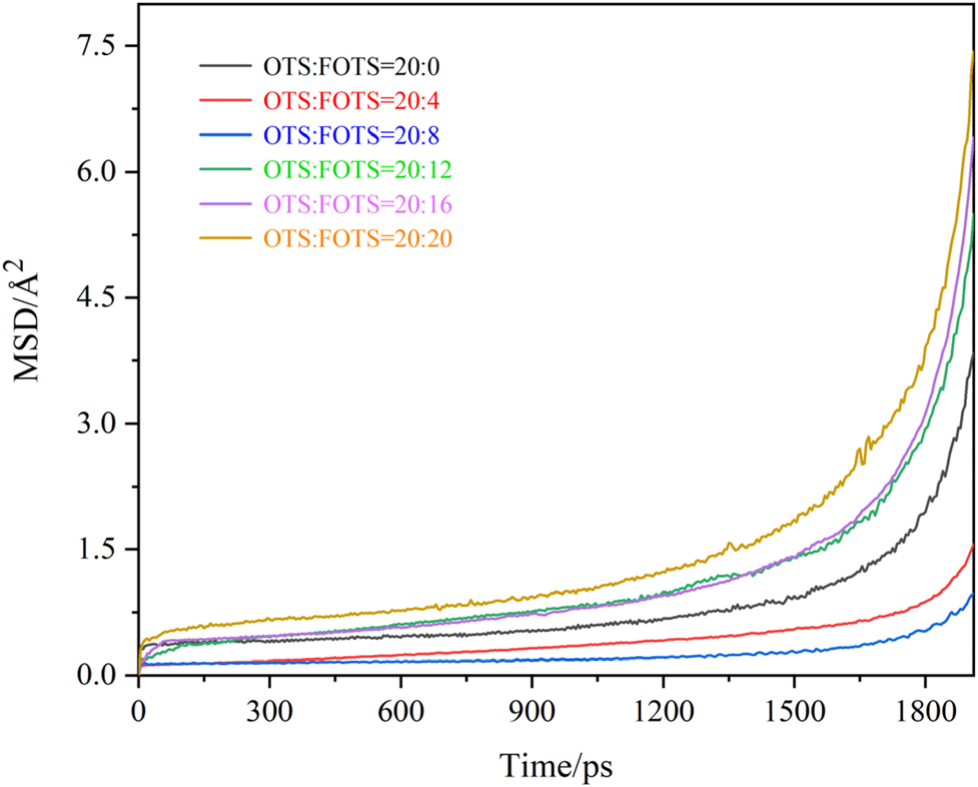
MSD curves of Cs atoms in OTS coatings modified with varying molecular chain numbers of FOTS.

The MSD curves of Cs atoms are shown in Fig. 6, and the calculated diffusion coefficients are summarized in Table 1. It can be seen that the MSD curves of Cs atoms exhibit three distinct phases. In the initial phase (0–25 ps), known as the ballistic regime, the cesium atoms experience negligible resistance from the surrounding medium, causing the MSD curve to increase sharply over a very short time period. The intermediate time interval of 25–1500 ps corresponds to the intermediate regime, where cesium atoms become temporarily confined in localized regions due to the combined influence of microstructural constraints, defect/interface effects within the OTS coating, and environmental conditions, resulting in a quasi-plateau behavior in the MSD curve. During the diffusion regime at time intervals of 1500–2000 ps, the system reaches a steady-state diffusion condition. The MSD curve of cesium atoms follows Einstein's theory of Brownian motion, with its slope being directly proportional to the diffusion coefficient. Compared to pristine OTS chains, the diffusion behavior of Cs atoms shows a nonlinear dependence on the FOTS chain content. With the increasing number of FOTS chains, the overall movement amplitude of the MSD curve exhibits a trend of initial increase followed by a decrease. When the number of FOTS chains is 8, the cesium atoms exhibit the smallest amplitude in the MSD curve, along with the minimum slope during the diffusion regime. Correspondingly, the diffusion coefficient initially decreases from 0.735 × 10^−6^ cm^2^ s^−1^ to 0.204 × 10^−6^ cm^2^ s^−1^ and subsequently increases to 2.053 × 10^−6^ cm^2^ s^−1^. The fundamental mechanism underlying this phenomenon lies in the formation of a dense superhydrophobic layer during FOTS cross-linking. As the FOTS chain content increases (below 40% proportion), the porosity of the OTS coating decreases, effectively hindering the diffusion of Cs atoms. However, the superhydrophobicity may be partially compromised due to excessive surface roughness when the FOTS chain proportion exceeds 40%, resulting in disordered molecular chain alignment and the formation of microcracks or pores. These structural defects create potential diffusion pathways for Cs atoms, leading to enhanced diffusion rates. Therefore, the optimal molecular chain number ratio is determined to be OTS : FOTS = 20 : 8.

**Table 1:** Diffusion coefficient of Cs atoms in FOTS-modified OTS coatings with varying FOTS molecular chain numbers

Ratio of OTS and FOTS	Diffusion coefficient (10^−6^ cm^2^ s^−1^)
20 : 0	0.735
20 : 4	0.298
20 : 8	0.204
20 : 12	1.343
20 : 16	1.657
20 : 20	2.053

### Effect of water molecules on the relaxation properties of cesium atoms

**Fig. 7:**
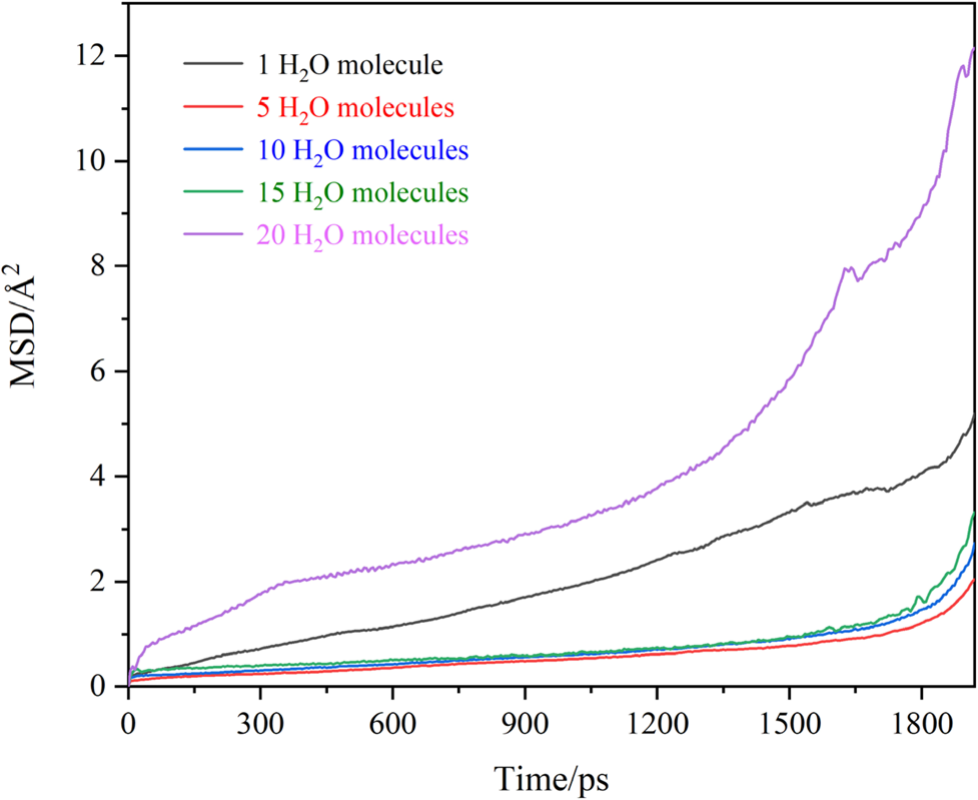
MSD curves of Cs atoms in OTS coatings modified with varying number of water molecules.

The formation process of OTS coatings potentially involves concurrent, interchain OTS condensation, interchain FOTS condensation, cross-condensation between OTS and FOTS chains, and chain-substrate condensation between polymeric chains and surface hydroxyl groups on the substrate. All these reaction pathways generate water molecules as byproducts. Under low-temperature conditions (maintained at 50 °C in MD simulations), residual water molecules (*n* = 1, 5, 10, 15, and 20 per simulation unit) may exert non-negligible effects on Cs atomic diffusion kinetics within the OTS coating. The system configuration maintained fixed stoichiometric ratios of 20 OTS chains and 8 FOTS chains, consistent with the optimized coating formulation. The MSD curves of Cs atoms are presented in Fig. 7, with the corresponding calculated diffusion coefficients in Table 2. It can be seen that the presence of water molecules exerts a significant influence on the diffusion process of Cs atoms. A marked enhancement in atomic displacement amplitudes is observed with increasing water content, correlating with progressive elevation of diffusion coefficients from 0.271 × 10^−6^ cm^2^ s^−1^ to 2.387 × 10^−6^ cm^2^ s^−1^. The presence of water molecules significantly increases the diffusion coefficient of Cs atoms due to two possible reasons. First, water molecules may react with molecular chains in the OTS coating to form hydroxyl functional groups, thereby inducing localized chemical modifications. This leads to regional swelling of the coating, which creates additional diffusion pathways for Cs atoms, ultimately resulting in an increase in the Cs diffusion coefficient. Second, water molecules exhibit strong polarity and form hydrogen bonds with hydroxyl groups in the coating. The presence of hydrogen bonds leads to coating expansion, increasing free volume and thereby enlarging diffusion pathways for Cs atoms. In addition, hydrogen bonds destabilize the original ordered arrangement of perfluorinated chains, significantly compromising the hydrophobicity of the coatings. Consequently, minimizing water content during the deposition of OTS anti-relaxation coatings on atomic cell walls is critical for effectively suppressing the diffusion of Cs atoms. Theoretically, the ALD technology can effectively reduce water molecules in anti-relaxation coatings. Conducting related research in this regard is of practical significance.

**Table 2:** Diffusion coefficient of Cs atoms in FOTS-modified OTS coatings with varying number of water molecules

Water molecules	Diffusion coefficient (10^−6^ cm^2^ s^−1^)
1	0.271
5	0.722
10	0.926
15	1.644
20	2.387

### Effect of temperature on the relaxation properties of cesium atoms

**Fig. 8:**
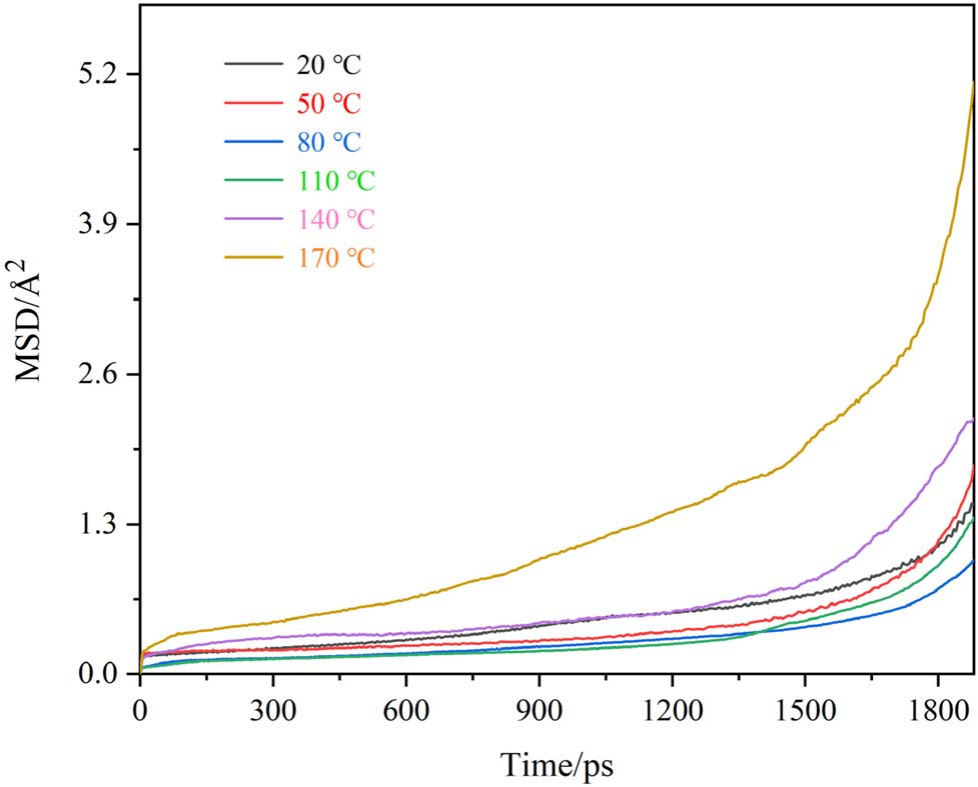
MSD curves of Cs atoms in modified OTS coatings at varying temperatures.

**Fig. 9:**
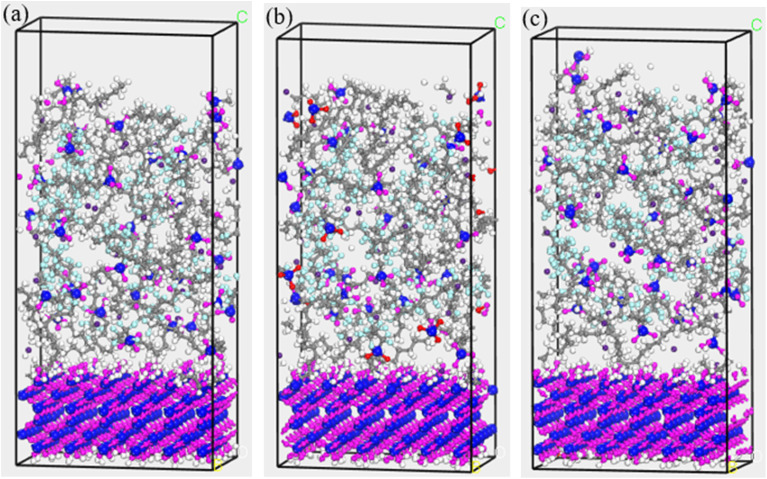
Structural evolution at three key temperature points: (a) 20 °C; (b) 80 °C; (c) 170 °C.

This section systematically investigates the temperature-dependent diffusion kinetics of Cs atoms in FOTS-modified OTS coatings. The methodological framework maintained fixed stoichiometric ratios of 20 OTS chains and 8 FOTS chains, with controlled residual water content (*n* = 1). Temperature gradients were established across six discrete points (20 °C, 50 °C, 80 °C, 110 °C, 140 °C, and 170 °C). The MSD curves of Cs atoms are presented in Fig. 8, with the corresponding calculated diffusion coefficients in Table 3. Fig. 9 shows the structural evolution at three key temperature points (20 °C, 80 °C, and 170 °C). It can be seen that the amplitude of the Cs atomic motion initially decreases and then increases as the temperature increases, with the corresponding diffusion coefficient decreasing from 0.344 × 10^−6^ cm^2^ s^−1^ to 0.172 × 10^−6^ cm^2^ s^−1^, and then increasing to 1.239 × 10^−6^ cm^2^ s^−1^. This phenomenon can be primarily attributed to two factors. First, temperature variations induce molecular chain conformational transitions. The incorporation of perfluorinated chains into the OTS chains substantially improves thermal stability and maintains the glassy state below 80 °C. This state significantly inhibits the diffusion process of Cs atoms. Under low-temperature conditions (<80 °C), moderate temperature elevation enhances the cross-linking degree of polymer chains, resulting in increased coating compactness that effectively suppresses the Cs diffusion process. This explains why the diffusion coefficient of Cs atoms decreases with increasing temperature below 80 °C. Second, continued temperature increase activates intensified chain segment motion and expanded free volume. This thermal activation may partially disrupt the cross-linked network of OTS chains and form structural defects within the coating, providing diffusion channels for Cs atoms, consequently leading to the observed increase in Cs diffusion coefficient at elevated temperatures.

**Table 3:** Diffusion coefficient of Cs atoms in FOTS-modified OTS coatings at varying temperatures

Temperatures (°C)	Diffusion coefficient (10^−6^ cm^2^ s^−1^)
20	0.344
50	0.422
80	0.172
110	0.281
140	0.610
170	1.239

## Conclusions

The self-assembly of FOTS-modified OTS molecular chains on the SiO_2_ (001) surface and the effects of FOTS amounts, water molecules, and temperature on the diffusion process of Cs atoms were studied in this work. The relevant conclusions are as follows.

(1) The film formation process of FOTS-modified OTS chains on SiO_2_ surfaces involves three distinct stages: initial anchoring, conformational rearrangement, and structural relaxation and equilibrium configuration. The molecular chains evolve from an initial perpendicular orientation into a final stable configuration parallel to the substrate surface. This revealed multistage assembly mechanism provides theoretical guidance for designing OTS-based anti-relaxation coatings.

(2) Increasing the FOTS content effectively reduces the diffusion coefficient of Cs atoms in the OTS coating. However, excessive FOTS chain content induces microcracks or voids in the film, leading to an accelerated diffusion rate. The optimum blending ratio of OTS to FOTS is determined to be 20 : 8, achieving a minimum diffusion coefficient of 0.204 × 10^−6^ cm^2^ s^−1^.

(3) The presence of water molecules exerts a significant influence on the diffusion process of Cs atoms. As the water content increases from 1 to 20, the diffusion coefficient rises from 0.271 × 10^−6^ cm^2^ s^−1^ to 2.387 × 10^−6^ cm^2^ s^−1^. Minimizing the water content during the deposition of OTS anti-relaxation coatings on atomic cell walls is critical for effectively suppressing the diffusion of Cs atoms.

(4) As the simulation temperature increases, the diffusion coefficient of Cs atoms exhibits a non-monotonic trend, initially decreasing and subsequently increasing. The diffusion coefficient reaches a minimum value of 0.172 × 10^−6^ cm^2^ s^−1^ at 80 °C. The FOTS-modified OTS chains substantially enhance the thermal stability of OTS anti-relaxation coatings, effectively suppressing Cs atom diffusion at 80 °C. This finding provides theoretical foundations for the subsequent fabrication of FOTS-modified OTS anti-relaxation coatings *via* the atomic layer deposition technology.

## Conflicts of interest

There are no conflicts to declare.

## Data Availability

The data that support the findings of this study can be obtained from the corresponding author. Hengjiao Gao, Email: gaohengjiao@163.com.

## References

[cit1] Du P., Yang F., Zhou Y., Quan W., Li J. (2025). Magnetic resonance linewidths independent with the laser linewidth in coherent population trapping (CPT) magnetometers. Measurement.

[cit2] Li R., Liu Y., Li J., Cao Q., Huang B., Zhai Y. (2024). Response signal enhancement for single-beam SERF atomic magnetometer with magneto-optical misalignment phenomenon. Opt. Laser Technol..

[cit3] Cao Q., Yu S., Xu L., Zhai Y. (2024). Spin-exchange relaxation-free magnetometer enhanced by biased weak measurement. Results Phys..

[cit4] Yang F., Li X., Du P., Quan W., Cui S., Gou W. (2025). Coherent population trapping (CPT) magnetometer empowering three-dimensional magnetic vector measurement based on dual-beam with lin//lin polarization. Measurement.

[cit5] Du P., Yuan J., Yang F., Huo X., Li J. (2024). A coherent population trapping magnetometer using a phase-delayed differential detection method. Opt Laser. Technol..

[cit6] Pischulti P., Duke T., Smith A., Klaus D., Amick R. (2024). Surveying and assessing ‘smart’ technologies to identify potential applications for deep space human exploration missions. Acta Astronaut..

[cit7] Dolgovskiy V., Lebedev V., Colombo S., Weis A., Michen B., Ackermann-Hirschi L., Petri-Fink A. (2015). A quantitative study of particle size effects in the magnetorelaxometry of magnetic nanoparticles using atomic magnetometry. J. Magn. Magn. Mater..

[cit8] Tang Y., Ip W., Yung K., Bi Z. (2024). Industrial information integration in deep space exploration and exploitation: Architecture and technology. J. Ind. Inf. Integr..

[cit9] Peng X., Liu E., Tian S., Fang L., Zhang H. (2022). Study of high-precision velocimetry technique based on absorption spectrum for deep space exploration. Acta Astronaut..

[cit10] Qiu S., Cao X., Wang F., Yue C., Zhang Z. (2019). Deep space exploration orbit design departing from circumlunar orbit of lunar base. Aerosp. Sci. Technol..

[cit11] Winterberg F. (2019). Nuclear microbomb propulsion for manned deep space exploration with return travel. Acta Astronaut..

[cit12] He Y., Shang W., Tan P. (2024). Insight into rechargeable batteries under extreme pressure and gravity for deep space exploration. J. Mater. Chem. A.

[cit13] Palaporn D., Tanusilp S., Sun Y., Pinitsoontorn S., Kurosaki K. (2024). Thermoelectric materials for space explorations. Mater. Adv..

[cit14] Taghavi M., Salary M., Mosallaei H. (2022). Multifunctional metasails for self-stabilized beam-riding and optical communication. Nanoscale Adv.

[cit15] Liu X., Hou X., Yan L., Zhang Y., Wang J. (2025). ZnO functionalized paraffin/diatomite phase change material and its thermal management mechanism in PDMS coatings. RSC Adv..

[cit16] Kim C., Choi M., Sim J., Kim H., Choi C. (2024). Area-selective atomic layer deposition (AS-ALD) of low temperature (300 °C) cobalt thin film using octadecyltrichlorosilane (ODTS) self-assembled monolayers (SAMs). Appl. Surf. Sci..

[cit17] Khalkhali S., Ranjbaran M., Mofidi D., Hamidi S., Tehranchi M. (2017). Improvement of the spin polarization lifetime in the ^85^Rb vapor cell by octadecyltrichlorosilane coating. Chin. J. Phys..

[cit18] Wang C., An D., Yang T., Gu L., Xie M., Lin X., Han C., Song Y., Deng Q., Wen F. (2024). Self-cleaning and amphiphobic properties of octadecyltrichlorosilane self-assembled modified polytetrafluoroethylene films. Colloids Surf., A.

[cit19] Wei Y., Gao S., Sun W., Wu X., Feng Y., Chu F. (2024). Anti-frosting and defrosting on photothermal superhydrophobic coatings based on silane hydrolysis and carbon nanotube doping. Appl. Therm. Eng..

[cit20] Latthe S., Demirel A. (2013). Polystyrene/octadecyltrichlorosilane superhydrophobic coatings with hierarchical morphology. Polym. Chem..

[cit21] Liu F., Yang H., Wang J., Qian Y., Wu J., Li S., Liu Q., Yang S., Xu S., Zhang X., Zhao Z., Wang J. (2019). Molecular interaction between asphaltene and quartz with different surface wettability: A combined study of experimental measurement and theoretical calculation. Fuel.

[cit22] Barriga J., Coto B., Fernandez B. (2007). Molecular dynamics study of optimal packing structure of OTS self-assembled monolayers on SiO_2_ surfaces. Tribol. Int..

[cit23] Hu L., Wang J., Hou K., Yang S. (2019). Robust ultralow friction between graphene and octadecyltrichlorosilane self-assembled monolayers. Appl. Surf. Sci..

[cit24] Xu H., Cao X., Chen X., Kong F., Liang H., Gao H., Xia H., Li J. (2024). First-principles study of the effect of oxygen vacancy and iridium doping on formaldehyde adsorption on the La_2_O_3_(001) surface. RSC Adv..

[cit25] Sethi S., Soni L., Shankar U., Chauhan R., Manik G. (2025). A molecular dynamics simulation study to investigate poly(vinyl acetate)-poly(dimethyl siloxane) based easy-clean coating: An insight into the surface behavior and substrate interaction. J. Mol. Struct..

[cit26] Li C., Zhou Z., Lin S., Meng X., Liu J., Zhou C. (2024). Studying how changes in the structure caused by hydrogen bonding affect the corrosion resistance of polybenzoxazine-based coatings using molecular dynamics simulations. Prog. Org. Coat..

